# COVID severity test (CoST sensor)—An electrochemical immunosensing approach to stratify disease severity

**DOI:** 10.1002/btm2.10566

**Published:** 2023-07-11

**Authors:** Sasya Madhurantakam, Jayanth Babu Karnam, Sriram Muthukumar, Shalini Prasad

**Affiliations:** ^1^ Department of Bioengineering The University of Texas at Dallas Richardson Texas USA; ^2^ EnLiSense LLC Allen Texas USA

**Keywords:** biosensors, COVID‐19, medical devices, nanobiology, patient‐targeted therapies, rapid diagnostics

## Abstract

With the evolution of the COVID‐19 pandemic, there is now a need for point‐of‐care devices for the quantification of disease biomarkers toward disease severity assessment. Disease progression has been determined as a multifactor phenomenon and can be treated based on the host immune response within each individual. CoST is an electrochemical immunosensor point‐of‐care device that can determine disease severity through multiplex measurement and quantification of spike protein, nucleocapsid protein, D‐dimer, and IL‐2R from 100 μL of plasma samples within a few minutes. The limit of detection was found to be 3 ng/mL and 21 ng/mL for S and N proteins whereas for D‐dimer and IL‐2R it was 0.0006 ng/mL and 0.242 ng/mL, respectively. Cross‐reactivity of all the biomarkers was studied and it was found to be <20%. Inter and intra‐assay variability of the CoST sensor was less than <15% confirming its ability to detect the target biomarker in body fluids. In addition, this platform has also been tested to quantify all four biomarkers in 40 patient samples and to predict the severity index. A significant difference was observed between healthy and COVID‐19 samples with a *p*‐value of 0.0002 for D‐dimer and <0.0001 for other proteins confirming the ability of the COST sensor to be used as a point of care device to assess disease severity at clinical sites. This device platform can be modified to impact a wide range of disease indications where prognostic monitoring of the host response can be critical in modulating therapy.

## 1 INTRODUCTION

COVID‐19 Pandemic has devastated the world with the emergence of the severe acute respiratory syndrome coronavirus (SARs‐CoV‐2). This outbreak has demonstrated the importance of on‐site diagnostic testing for emerging and re‐emerging diseases. Biomarkers for early disease detection and prognosis were needed to develop better treatment strategies for patients with severe coronavirus disease. The development of clinical biomarkers to assess disease severity and predict the clinical efficacy of drugs is urgently needed.^1^ Affordable, highly selective, and sensitive biosensors can contribute to the development of rapid and accurate tests on a large scale. Excellent research efforts have been made to develop serological and molecular diagnostic methods for accurate and early detection of disease onset. Despite efforts to control COVID‐19 infection, new severe acute respiratory syndrome coronavirus 2 (SARS‐CoV‐2) variants with different mutations continue to emerge and drive the coronavirus pandemic (COVID‐19). Several reports indicate that COVID‐19 patients present with severe symptoms after infection and often require disease surveillance to determine the possibility of re‐infection and to assess risk.^2^ Accurate quantification of clinical biomarkers provides a clear understanding of effective treatment strategies. This has been determined as the strategy for evidence‐based management of disease severity.^3^ This approach of utilizing host response for managing infectious diseases has been employed for sepsis management.^4^, ^5^

In studying the host response to this disease, viral load determination helps facilitate decisions for personalized medications that effectively reduce the risk of common treatment strategies. Antibody‐based approaches help to provide sufficient information to clinicians. Selective determination of other biomarkers of COVID‐19 could lead to effective treatment strategies which asses the clinical progress in a very short time. Biomarkers could serve as clinical trademarks of COVID progression and severity indication.^6^ Coronaviruses are comprised of single‐stranded positive‐gene RNA covered with RNA‐dependent RNA polymerase gene (RdRP) and four structural proteins named spike surface glycoprotein (S), an envelope protein (E), matrix protein (M) and nucleocapsid protein (N). These two proteins serve as structural target sites and can be effectively useful for the detection of coronavirus‐induced infections.^7^ These proteins are immunogenic and trigger the immune response by producing immunoglobulins against coronavirus. Identification and quantification of these immunoglobulins might help in the early detection of disease.^8^, ^9^, ^10^ Therefore, this spike and nucleocapsid proteins could be potential targets for early diagnosis of COVID‐19. The spike protein, located on the surface of viral particles, is highly immunogenic. The nucleocapsid protein interacts with the membrane protein during virion assembly and forms complexes with genomic RNA. It plays an important role in the transcription and translation of viral RNA and interferes with the cell cycle process of host cells, confirming its multifunctionality.^11^ Thus, these two proteins have been shown to play a crucial role in the COVID‐19 infection process. Several antibody‐based assays methods have been described for the quantification of these two proteins.^12^, ^13^, ^14^

Due to the invasion of these virus particles inside the cells, affected individuals usually develop symptoms 2–14 days after infection. In immunocompromised patients, the risk is lower compared with healthy individuals because of less immune response.^15^ Cytokine storm is defined as the quick release of many cytokines into the bloodstream due to immune reactions triggered by infections. These hypercytokinemic conditions lead multiple to organ failure and death. This can be identified by measuring the neutrophil‐to‐lymphocyte ratio in the blood. Complex analysis was reported by the researchers for the in‐depth analysis and to understand the pathophysiology of cytokine storm.^16^ Accurate quantification of clinical biomarkers provides a clear understanding of effective management & treatment strategies. Elevated proinflammatory cytokines (IL‐2, IL‐8, IL‐17) were meagerly controlled by anti‐inflammatory cytokines (IL‐1, IL‐4, IL‐10, IL‐11, IL‐13) which influence the degree of coagulation.^16^

D‐dimer has been implicated as an important protein biomarker in assessing disease severity as it is a biomarker of fibrinolysis.^17^, ^18^, ^19^, ^20^ Recent studies show that elevated D‐dimers are one of the causative factors for increased mortality in COVID‐19 patients.^21^, ^22^ Because of their unclear role in regulating coagulation during pathological events, abnormal D‐dimers are associated with many disease states such as sepsis, myocardial infarction, disseminated intravascular coagulation (DIC), and so on.^17^ D‐dimer can also be associated for deep vein thrombosis, venous thromboembolism, DIC, stroke, and thrombosis in cancer and acute pulmonary embolism.^23^, ^24^, ^25^, ^26^, ^27^, ^28^ Although elevated D‐dimers have not been found in all patients with COVID‐19, they are commonly classified as a biomarker of prognosis and identified as an important risk factor for fatal organ injury.^29^ The correlation of D‐dimer with clinical staging has been reported strongly to identify the disease severity stages depending on the D‐dimer levels.^17^ D‐dimer has been reported as an accurate biomarker to predict mortality rate depending on its value with a cutoff value of 1.5 μg/mL upon admission.^30^ Therefore, the measurement of D‐dimer levels provides important information about the prognosis of COVID‐19 and helps physicians to initiate immediate therapies.^31^

IL‐2R alpha is another interleukin that was aggressively elevated in COVID patients who had severe disease.^32^ This circulating receptor has been shown to regulate the activation of T lymphocytes, leading to life‐threatening multi‐organ failure.^33^ Reports indicate that the increase in IL‐2R levels are related to disease severity.^34^, ^35^, ^36^ Comprehensive immunological studies have reported cytokine expression in mild and severe COVID‐19. In these reports, it was mentioned that patients with mild or moderate disease showed lower expression of cytokines, whereas patients with severe COVID‐19 showed extremely higher expression of cytokines, as shown in.^37^ Therefore, IL‐2R could serve as a potential biomarker for early prediction of COVID‐19 disease progression. Higher IL‐2R levels have also been associated with longer disease duration.^32^, ^34^ Traditional methods for quantifying these biomarkers include enzyme‐linked immunosorbent assay (ELISA) and chemiluminescence techniques. These methods would not provide just‐in‐time information required for rapid turnaround time between reporting and changes to therapy.

Electroanalytical techniques have been utilized to design sensors for applications such as for sensitive detection of SARS‐CoV‐2 spike glycoprotein using molecularly imprinted polypyrrole.^38^ Screen‐printed electrode modified with peptide was utilized for measuring spike protein concentration in 15 min.^39^ Electrochemical biosensors for the detection of SARS‐C0V‐2 RNA^40^, ^41^ spike protein from nasal swabs,^42^ and saliva^43^ were reported. All these methods could detect a single biomarker for disease detection. An electrochemical immunosensor for the detection of spike protein was reported by utilizing gold nanostructures. This low‐cost immunosensor developed using screen‐printed electrodes is capable of detecting spike protein 9 s from 10 μL of saliva samples.^44^ Multiplex detection of SARS‐CoV‐2 with several mutants was reported by Helena et al. by utilizing a CRISPR‐based point‐of‐care device.^45^ COVID‐19 detection using a Geno sensor utilizing data and machine learning techniques were reported.^46^ Label‐free detection of nucleocapsid protein was reported by Elif et al. utilizing electrochemical methods.^47^ Multimarker evaluation was presented by a few research groups. Biotin‐labeled SARS‐CoV‐2 RBD antibody was utilized for the simultaneous detection of IgG and IgM antibodies.^48^ Point‐of‐care devices based on immunosensing methods that could detect spike protein as well as IgG antigen^49^ and electrochemical voltammetric sensors utilizing CRISPR‐Cas12a^50^ were developed. These point‐of‐care testing methods help in the treatment of early‐stage patients and are not sufficient for disease monitoring and severity prediction. Label‐free and multimarker evaluations are necessary for continuous monitoring of COVID‐19. Nano biosensors are promising tools for the detection of coronavirus by utilizing aptamers, genomic materials, and optical and superparamagnetic approaches.^51^, ^52^ To address these scenarios, we present our research on COVID‐19 detection, severity prediction, and personalized management using a rapid biosensor prototype. These multi‐analysis systems could give us better insight into disease progression and severity. Given the urgent need for the detection of COVID‐19 biomarkers, here we have developed an ideal strategy to detect the disease and measure its severity with a single test. To develop the proposed CoST sensor, EnliSense designed a rapid electroanalytical instrument platform. The use of this cost‐effective and rapid test will enable clinicians to provide personalized treatment strategies for continuous disease monitoring. With respect to the clinical sample testing scenario, the innovation is focused on demonstrating the multiplex measurements of four biomarkers using a single sensor which would be more cost‐effective as compared to the conventional methods which are in clinical use that require the use of multi‐well plates.

## 2 MATERIALS AND METHODS

### 2.1 Reagents

Sterile‐filtered phosphate buffer saline (PBS) with MgCl_2_ and CaCl_2_, dithiobis (succinimidyl propionate) (DSP, >95% purity), dimethyl sulfoxide (DMSO) (99.9% purity), and Tween‐20 surfact‐amps detergent solution (10% w/v solution) were purchased from Thermo Fisher Scientific, USA. Spike protein, nucleocapsid protein, and the corresponding antibodies were purchased from Sino Biological US Inc, and D‐dimer, IL‐2receptor were purchased from Abcam along with the corresponding antibodies. Luminex kits for 20plex were purchased from R&D Systems, Inc, and MSD kits for spike protein, nucleocapsid protein, and IL‐2R were purchased from Meso Scale Diagnostics, LLC. Healthy human pooled plasma (30 pools, blood‐derived) was purchased from Innovative Research, Inc (USA). COVID‐19‐positive plasma samples from COVID‐positive patients were purchased from the COVID‐19 biorepository, University of Texas Southwestern. All these proteins, antibodies, and patient samples were stored according to their respective storage instructions until further use. All antibodies were diluted in PBS, and various antigen concentrations were prepared in healthy pooled human plasma to generate a calibrated dose–response curve and to perform other sensor property studies. To avoid protein denaturation, freeze–thaw cycles were limited to less than three.

### 2.2 Characterization of materials

#### 2.2.1 FTIR analysis

Fourier‐transformed infrared spectroscopy is used to confirm the surface functionalization of the electrodes. The absorption spectra of all functionalization steps were recorded using a Nicolet iS50 FTIR spectrometer (Thermo Scientific Inc.). The glass substrate was coated with gold by sputtering to mimic the electrode surface. A thin ZnO layer of about 100 nm was deposited on the gold substrate under controlled conditions of 85% argon for about 60 min at a speed of 8 rpm. 10 mM of DSP dissolved in DMSO was incubated on the Au/ZnO surface and then the FTIR spectrum was recorded. Then DSP was removed, and the surface was functionalized with the antibodies prepared in PBS solution α S protein, α N protein, α D‐dimer, and α IL‐2R for about 2 h. Parameters were kept constant for all measurements, with a resolution of 4 cm^−1^, 256 scans, and a spectral range between 400 and 4000 cm^−1^. The reference background of air was measured before recording each spectrum.

#### 2.2.2 Measurements of the zeta potential

The measurement of the zeta potential (ZP), (ζ) provides information about the charge state. It measures the electrophoretic mobility in buffer solutions. The ZP was measured using the Malvern Zetasizer NanoZS (Malvern Instruments, UK). ZP for all four antibodies α S protein (100 μg/mL), α N protein (100 μg/mL), α D‐dimer (20 μg/mL), and α IL‐2R (10 μg/mL), prepared in PBS solution. To test the stability of the antibody–antigen interactions, the antibodies were mixed with their antigens at low (58 ng/mL, 58 ng/mL, 10 ng/mL, and 0.05 ng/mL of S,N‐proteins, D‐dimer and IL‐2R, respectively) and high (100 μg/mL, 100 μg/mL, 10 μg/mL ng/mL and 53.09 ng/mL of S,N‐proteins, D‐dimer and IL‐2R, respectively) concentrations and the ZP was measured.

### 2.3 Design of biosensor experiments

Biomarker identification and quantification through a portable and rapid detection method were developed. This CoST sensor measures biomarkers in blood matrices within minutes with an incubation time of 15 min at very low sample volumes (25 μL for each biomarker, 100 μL for total samples). The biosensor was designed to measure spike protein, nucleocapsid protein, d‐dimer, and IL‐2receptors from plasma samples to differentiate between COVID −Ve and COVID +Ve patients and estimate disease severity. A calibrated dose response was developed using spiked plasma samples, and recovery was estimated from a linear graph. Biosensor properties such as dynamic linear range, accuracy, sensitivity, selectivity, cross‐reactivity, and interference were tested for all biomarkers. This CoST sensor was also validated on 40 clinical samples (20 healthy and 20 COVID‐19 positive). Healthy, positive with mild disease, and positive with severe disease were classified using the developed CoST sensor, and the results were compared with the reference standard using chemiluminescence and Luminex techniques. All these experiments were conducted at room temperature, and we recorded temperature while conducting experiments. The variance in the temperature is between 21 and23°C for all the experiments. We have not studied the effect of variation in extreme high and low temperatures as the designed sensor will be used only in ambient controlled temperatures even in the clinical study. As the real‐time testing of the developed CoST sensor will be in plasma samples or other blood matrices, all the experiments were conducted in healthy human pooled plasma samples. Calibrated dose responses were also calibrated using pooled healthy human plasma samples to make the biosensor more efficient in capturing specific analytes in the presence of other commonly existing proteins in blood matrices such as plasma (Figure 2). All the chemicals and antibodies used in the study were non‐toxic. In case of DMSO and Tween‐20 (superblock), it has very low toxicity. A number of animal studies as well as cell studies have been performed that have shown mild responses.^53^ This is a standard reagent in sensor assay development and has been widely used for biochemical sensor assay development. With regards to healthy human pooled plasma samples, all the samples were viral and bacterial tested, laboratory‐grade certified, and not infectious. COVID‐19 plasma samples were procured from a repository, and they were heat inactivated at 56°C for 30 min. To ensure the absence of the contagion COVID‐19 samples, were heat‐inactivated upon receipt at 56°C for 30 and stored the samples for further use in the study. All the samples were handled inside BSL‐2 Biosafety cabinet. While handling samples and conducting experiments, we have used personal protective equipment such as N‐95 mask, arm‐length gloves, 70% ethanol, disposable lab coat, goggles, face shield, head, and foot covers to ensure the handling person is well protected. Before and after handling the samples, UV light was turned on for 60 min in the Biosafety cabinet.

### 2.4 Biosensor preparation and electrochemical measurements

The CoST sensor consists of a gold working electrode and a gold reference electrode. The surface of the working electrode was modified with ZnO, a semiconductor material that improves the surface‐to‐volume ratio of the working electrode. A thin film of about 100 nm was deposited on the electrode surface by sputter deposition, a unique physical deposition method that enables uniform development of the nanomaterial layer on the substrates with better adhesion properties. The electrochemical impedance measurement is one of the most important biosensor techniques for measuring the interactions of biomolecules. As described in the previous work of our group,^54^ the device consists of a sensor array, a reader, and integrated software. The sensor array consists of 16 working electrodes surrounded by a reference electrode to rapidly measure the impedance of each of the electrodes simultaneously. To maintain the electrochemically active surface area constantly across the electrodes, ZnO deposition conditions and deposition time (60 min) along with DSP crosslinker (10 mM, 90 min) and antibody incubation time (90 min) during immunoassay were kept constant across all the electrodes for all the experiments in this study.

### 2.5 Development of immunoassay

To immobilize DSP on the ZnO surface, 10 mM of DSP was dissolved in DMSO and incubated for 1 h 30 min at room temperature (Figure S1: step 1). Extreme precautions were taken when preparing the DSP solution in DMSO because it is light‐sensitive and hydrolyzes easily. After incubation, DSP was removed and then incubated with antibodies. The NHS ester group on the surface of the electrode provides space for the amino‐reactive surface to form an amine bond with the amino group of the antibodies. Specific antibodies such as α S protein (100 μg/mL), α N protein (100 μg/mL), α D‐dimer (20 μg/mL), and α IL‐2R (10 μg/mL) were prepared in PBS and functionalized on specific electrodes (Figure S1: step 2) to capture their specific proteins (S‐protein, N‐protein, D‐dimer, and IL‐2R). After the successful functionalization of the antibodies, Superblock was used to hydrolyze and unbind the junction sites (Figure S1: step 3) to avoid nonspecific antigen interactions (Figure S1: step 4). Tween‐20 surfact‐amps detergent solution (10% w/v solution) was used as a super block. A total of 10% w/v solution of Tween‐20 in PBS solution was added to about 100 μL of the sensor surface and incubated for 10 min. After incubation, the solution was removed and washed with PBS to ensure the excess amount of superblock is removed and used for further experiments. All these assay steps are shown in Figure S1. Between each step, PBS was used for washing to remove unbound or excess molecules.

A wide linear range was chosen to develop a calibrated dose–response curve for all proteins. Various dose concentrations were prepared at four‐fold dilutions in pooled human plasma. The percent change in Zmod compared to the blank (pooled human plasma) was calculated for each dose and used to generate a linear curve. Sensing parameters such as limit of detection (LOD) and limit of quantitation (LOQ) were calculated for each biomarker using the calibrated dose–response curves (Table S1). Studies on biosensor characteristic properties were also performed in pooled human plasma samples, such as cross‐reactivity and specificity. Each biomarker was tested with three other biomarkers to generate a cross‐reactivity study. Specificity and interference were tested by using a cocktail of nonspecific biomarkers compared with a specific biomarker in the absence and presence of the specific biomarker. All measurements were performed in triplicate (*n* = 3). To reduce the interference of the components that exist in the blood matrices, we optimized the sensor performance in pooled healthy human plasma samples. Sensor performance evaluation such as calibrated dose responses, cross‐reactivity, and specific studies also performed in pooled healthy human plasma samples (Figures 2, 3, 4, 5). As we have used antibodies to modify the sensor surface, after preparation sensors should be stored at 4°C until further usage. Common degradation factors for antibody includes high temperature, frequent or multiple freeze–thaw cycles, pH variation such as too low or too high pH, oxidation, glycation, etc. If we store the sensors at room temperature for a long time it might impact the sensor performance. We have not done any extensive studies on the long‐term performance of the developed sensors yet, but we would consider them for our future studies. All the sensors prepared, and solutions used were discarded time to time in biosafety‐hazard bins. The sensors used for COVID‐19 samples and COVID‐19 plasma samples and pipette tips used were added into bleach solution for 30 min and then discarded in biohazard bins.

### 2.6 Data visualization and statistical analysis

All data are presented as mean ± SEM, *n* = 3 unless otherwise noted. Statistical representation followed as ns: not significant, *p 0.05, **p 0.01, ***p 0.001, ****p 0.0001. Percent covariance was less than 20% variance in accordance with CLSI standards. The calibrated dose response was analyzed by asymmetric nonlinear fitting (five parameters). One‐way ANOVA was performed to measure the difference in significance between specific and nonspecific biomarker interactions. Nonparametric unpaired Mann–Whitney tests were used to compare the healthy and COVID‐19 groups. All statistical calculations comparing healthy and COVID‐19 cohorts were performed using GraphPad Prism. One sample *t*‐test and Wilcoxson test were used to compare the results obtained with CoST and Luminex. The Bland–Altman test was performed to measure differences between Luminex and CoST sensor. The area under the curve (AUC) was measured by the receiver operator characteristic curve (ROC) using the Wilson/Brown test with a 95% confidence level to evaluate the specificity and sensitivity of the developed CoST sensor.

### 2.7 Procurement of human subject samples

As per the reference article,^55^ a study with effect size of 1 with a power of 0.8, alpha of 0.05, the sample size should be 30. In our study, we have used a total of 40 samples (20 healthy and 20 COVID‐19) which are sufficient to determine statistical significance between healthy and COVID‐19 cohorts. We have used 20 healthy plasma samples and 20 COVID‐19‐positive plasma samples in this study. All the healthy plasma samples were purchased from BocaBiolistcs LLC. These samples were not heat‐inactivated and processed in Biosafety level 2 as recommended by BocaBiolistics LLC and the package has been received from BocaBiolistics according to IATA regulations. A total of 20 COVID‐19 positive human plasma samples were purchased from COVID‐19 Biorepository, University of Texas at Southwestern (UTSW), based on a minimally reviewed IRB approved by the Human Subjects Research Office, University of Texas at Dallas titled “Evaluation of multiplexed biomarkers for COVID‐19 detection (IRB‐22‐328)”, under the IRB titled “de‐identified plasma samples from COVID patients together with deidentified data set collected under IRB # STU 2020‐0375”. All these samples were heat‐inactivated before we received them. After receiving, the samples were processed inside the biosafety cabinet according to COVID‐19 sample handling protocols and instructions. We have the demographic and sex information for the COVID‐19‐positive samples, but we do not have the healthy plasma sample demographic and sex information. So, the results presented in the current study were not interpreted using demographics, age, and sex.

## 3 RESULTS AND DISCUSSION

### 3.1 FTIR analysis

Fourier transform infrared spectra were used to understand the mechanism of interaction of antibody and crosslinker. To develop a robust biosensor, it is important to establish a stable immunochemistry on the surface of the electrode. The FTIR spectra of the functionalized DSP and antibody‐immobilized surfaces are shown in Figure S2. As shown in Figure S2A, the spectrum was recorded in the wavelength range of 500–3100 cm^−1^. The functionalization of DSP on the ZnO surface was confirmed by the presence of thiol bonds around 2900–3100 cm^−1^. The peak around 700 cm^−1^ indicates the presence of the methylene mixture in DSP. The peak at 1026 cm^−1^ confirms the presence of esters in DSP, whose intensity decreased after antibody immobilization. The peak observed at 1320 represents the symmetrical C–N–C stretch of NHS in DSP. The double peaks observed at 1421 and 1441 cm^−1^ confirm the deformation of the methylene (CH_2_) shears in DSP. The peaks at 1675 and 1756 cm^−1^ indicate the presence of the carbonyl stretching of NHS and ester, respectively. The peaks at 2931 and 3015 ^−1^ indicate the presence of saturated and unsaturated C–H stretching, respectively. This confirms the binding of DSP to ZnO. In addition, the mechanism of antibody conjugation was verified. The binding of the antibody to DSP occurs by aminolysis, a process in which the C=O bond of the NHS ester in DSP is broken. This was confirmed by the disappearance of the peak at 1756 cm^−1^ in the spectra of all four antibodies (Figure S2B–E). The shift of the peak from 1675 cm^−1^ to 1638–1640 cm^−1^ was observed with increased intensity, confirming the immobilization of the antibody on the ZnO surface by DSP through the formation of an amide bond.

### 3.2 ZP characterization of study proteins

**FIGURE 1:**
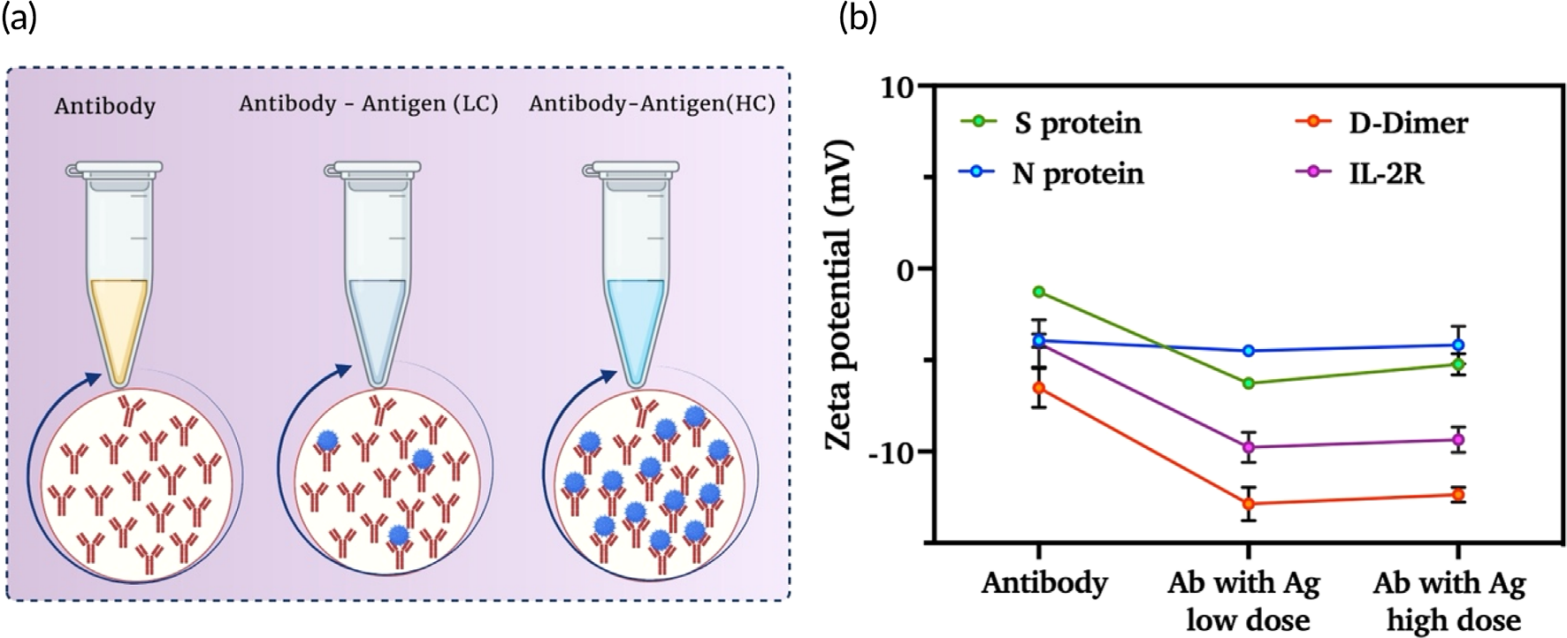
(a) Pictorial representation of antibody, antibody–antigen binding efficiency representation at low and high concentrations of antigens. (b) Zeta potential values for antibodies as well as for antibody–antigen conjugates at low and high concentrations of antigens (*n* = 3) (Created using Biorender.com).

The measurement of ZP provides information about the surface charge and stability of proteins. Under an applied electric field, proteins are surrounded by counterions and co‐ions and enter a neutral charge state. This charge state or ZP of a protein can be calculated by measuring the electrophoretic mobility of proteins. Antibody, antibody, and antigen mixture (low and high concentrations) were taken for ZP measurements as represented in Figure 1a. Figure 1b shows the change in ZP from antibody solution to antigen–antibody interaction at low and high concentrations. All four antibodiesα S protein, α N protein, α D‐dimer, and α IL‐2R have a stable ZP of <7 mV. Antigen–antibody interactions and low and high concentrations did not have a major effect on the ZP/charge of the antibodies. The ZP of αS protein antibodies conjugated with S protein was −6.2 mV at low concentrations and −5.2 mV at higher concentrations. The ZP of α n‐protein antibody was ~ −4 mV and that of the D‐dimer was ~ −12 mV at both low and high antigen concentrations, demonstrating their high stability. αIL‐2R showed a ZP value of −8 mV at low antigen concentration and − 12 mV at high antigen concentration. These results indicate that the antibodies are stable and have a constant charge when interacting with different antigen concentrations.

### 3.3 CoST sensor for the quantification of biomarkers toward COVID‐19 identification and severity

**FIGURE 2:**
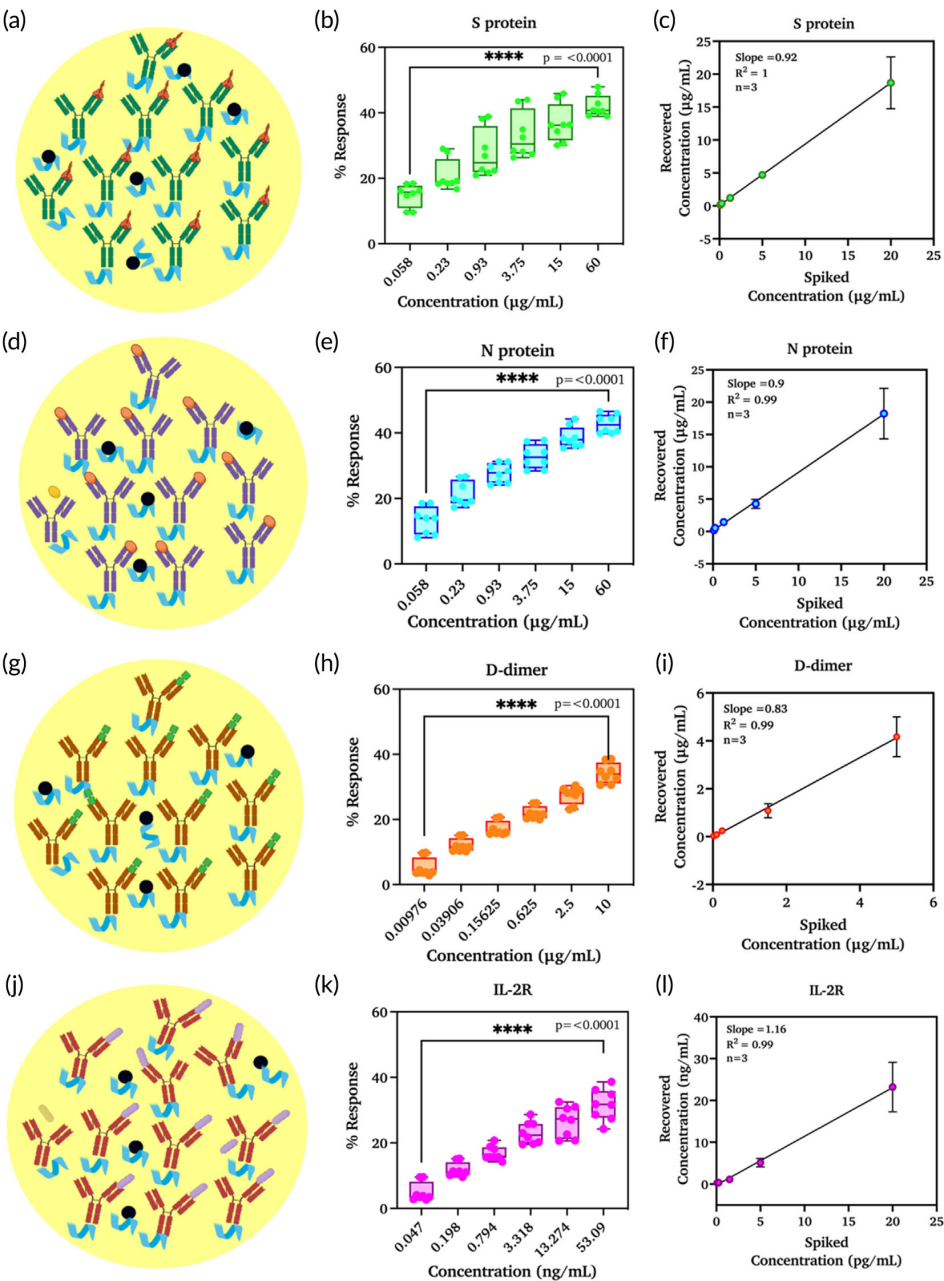
Pictorial representation of electrode surface for spike protein (a), nucleocapsid protein (d), D‐dimer (g), and IL‐2R (j). Calibrated dose responses (*n* = 8) for spike protein (b), nucleocapsid protein (e), D‐dimer (h), and IL‐2R (k). Graph showing the regression analysis for spiked concentrations versus recovered concentrations (*n* = 3) for spike protein (c), nucleocapsid protein (f), D‐dimer (i), and IL‐2R (l). (Created using Biorender.com (a, d, g, j)).

We have selected four biomarkers such as spike and nucleocapsid proteins for identification and D‐dimer and IL‐2R for severity prediction. All these four biomarkers were tested on the sensor to develop a successful CoST sensor. ZnO thin film‐coated gold electrodes were used to immobilize antibodies against each specific biomarker as described in the immunoassay development Section 2.5. Surface modification of the working electrode with ZnO, a semiconductor material, improves the surface coverage of the electrode, which enables the binding of a huge fraction of biomolecules. Pictorial representation of functionalized electrode surface for detecting specific targets was shown for each of the biomarker in the Figure 2a, d, g, j.

Electrochemical impedance measurements were performed over the electrodes at various frequencies. A calibrated dose response was measured for all four biomarkers considering their physiological ranges. A dynamic range of 58 ng/mL–60 μg/mL was chosen for spike protein and nucleocapsid proteins, whereas the range for D‐dimer was 10 ng/mL–10 μg/mL, and for IL‐2R was 0.05 ng/mL–53.09 ng/mL. All dose–response ranges covered both the elevated and very low dynamic ranges that can occur under normal and pathological conditions. The dose–response range for each of these biomarkers was strung to identify healthy, mildly impaired, and severely impaired patient cohorts based on this dynamic range. Spike protein showed a wide dynamic range from 58 ng/mL to 60 μg/mL (Figure 2b) with a specific signal threshold (SST) of 2.67%, which was much lower than the signal response measured for the lowest concentration. SST is a robust indicator of the sensor performed and is considered as a minimum signal required by the sensor for the target analyte. SST was calculated by measuring the average of blank sample response and multiplied by three times. The limit of detection and limit of quantitation for spike protein was calculated based on the SST value and were 0.049 μg/mL and 0.065 μg/mL, respectively. The nucleocapsid protein showed a similar dynamic response as the spike protein in the range between 58 ng/mL and 60 μg/mL (Figure 2e). The limit of detection was calculated to be 0.003 μg/mL, whereas the limit of quantitation was 0.021 μg/mL in spiked plasma samples because of the very low SST of 2.72%. Both proteins play an important role in eliciting the host's immune response and are abundantly expressed during infection.

However, electrochemical methods for the detection and quantification of these antigens were very limited. The signal measured with electrochemical impedance is proportional to the affinity of the biomarker for the antibody on the functionalized sensor surface. A dose‐dependent signal response was also observed for D‐dimers and nucleocapsid proteins. D‐dimer showed a dynamic range of 0.05 ng/mL–10 ng/mL, whereas the dynamic range of IL‐2R was 0.047 ng/mL–53.09 ng/mL (Figure 2h, k). It is well known that D‐dimer and IL‐2R are important moderators associated with disease severity. The increase in D‐dimer and IL‐2R levels correlated with the onset of disease progression. A high mortality rate also corresponded with increased D‐dimer and IL‐2R levels (48–50). The LOD was calculated as 0.0006 ng/mL for D‐dimer and 0.242 ng/mL for IL‐2R. The limit of quantification was calculated as 0.016 ng/mL for D‐dimer and 0.264 ng/mL for IL‐2R, with SSTs of 3.71% and 3.92%, respectively. In addition to the wide linear range and low detection limit, each biomarker showed a significant statistical difference between concentrations, as confirmed by one‐way ANOVA with 95% confidence intervals. The significant difference was calculated as described in this section. This confirms that the developed CoST sensor is highly sensitive to all four biomarkers over the entire dose–response range, which facilitates the discrimination and categorization of patients into healthy, moderately affected, and severely affected.

After the successful establishment of vigorous calibrated dose responses for all biomarkers, the performance of the sensor to estimate unknown concentrations in pooled human plasma was validated by performing spike and recovery studies. Briefly, known biomarker concentrations were spiked individually into plasma, and the concentrations were estimated from the calibrated dose–response curve generated. All measurements were performed in triplicate for each biomarker, and the results are shown in Figure 2c, f, i, l. The spiked concentrations were represented as actual concentrations on the *x* axis, and the measured concentrations were presented as calculated concentrations on the *y* axis. A regression coefficient of 0.99 and slope values of 1.074 (S protein), 0.9 (N protein), 0.829 (D dimer), and 1.166 (IL‐2R) were measured. This confirms the ability of the sensor to determine the unknown concentrations of all biomarkers in plasma samples.

**FIGURE 3:**
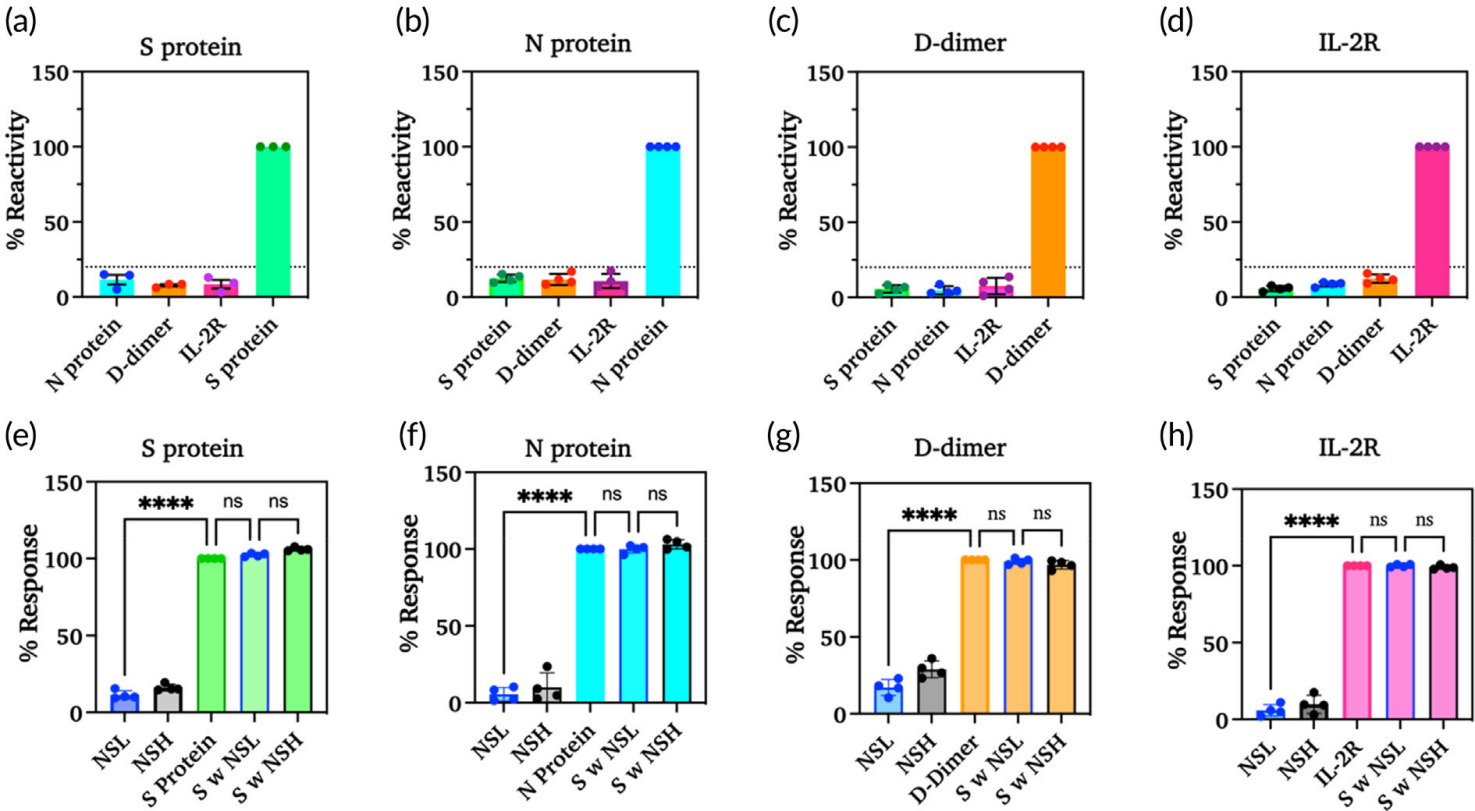
Cross‐reactivity (a)–(d) and specificity (e)–(h) of the developed CoST sensor in the presence and absence of a nonspecific biomarker (*n* = 4).

Electrochemical biosensors have received more recognition in the development of point‐of‐care devices due to their high sensitivity and specificity. The designed CoST sensor was evaluated for its specificity and selectivity toward individual biomarkers to confirm its efficiency. Assessment of sensor performance is further reliable when it can specifically recognize the target biomarkers in the presence of supplemental biomarkers. To endorse the specificity performance of the CoST sensor, each sensing electrode was tested with a cocktail fusion of nonspecific biomarkers at low and high concentrations, represented as non‐specific‐low (NSL) and non‐specific‐high (NSH) as indicated in Figure 3e–h. To evaluate the effects of interference while detecting specific biomarkers in the presence of nonspecific biomarkers, a similar study was performed. However, in this study, each specific biomarker (S) was assorted with nonspecific biomarkers at low (S w NSL) and high (S w NSH) concentrations in equal proportions, and the sensor performance to measure each specific biomarker was evaluated without interference (Figure 3e–h). The specificity of the sensor was confirmed by performing a one‐way ANOVA to measure the statistical difference between specific and nonspecific biomarkers at low and high concentrations in the absence and presence of the specific biomarker. A significant difference ****p < 0.0001 was measured for specific biomarkers compared with NSL and NSH conditions. A nonsignificant (ns) difference was measured for the detection of specific biomarkers together with nonspecific biomarkers at both low and high concentrations. These results confirm that the ability of the sensor to give false‐positive results is reduced by its high specificity for rapid measurement of the target biomarker in biological fluids without interfering effects. The selectivity of the sensor was evaluated by performing a cross‐reaction study with each biomarker. Precisely, each target biomarker was cross‐validated with other nonspecific biomarkers one after the other. As shown in Figure 3a–d all sensors designed to measure target biomarkers showed 100% reactivity to target biomarkers, while sensor reactivity to nonspecific biomarkers was <20%. This study aimed to estimate the selectivity of the biosensor for individual biomarkers by measuring the target biomarker under pragmatic conditions where the concentrations of the nonspecific biomarkers may be higher than that of the specific biomarker. These results confirm the selectivity of the biosensor for the target biomarker.

**FIGURE 4:**
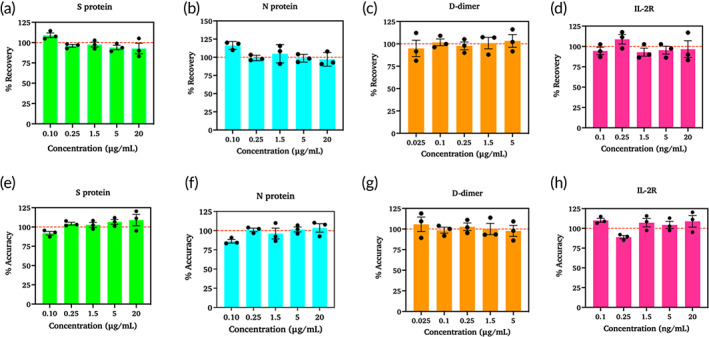
Shows the ability of the CoST sensor to detect unknown spiked concentrations in terms of its recovery rate (a)–(d) and accuracy (e)–(h) (*n* = 3).

**FIGURE 5:**
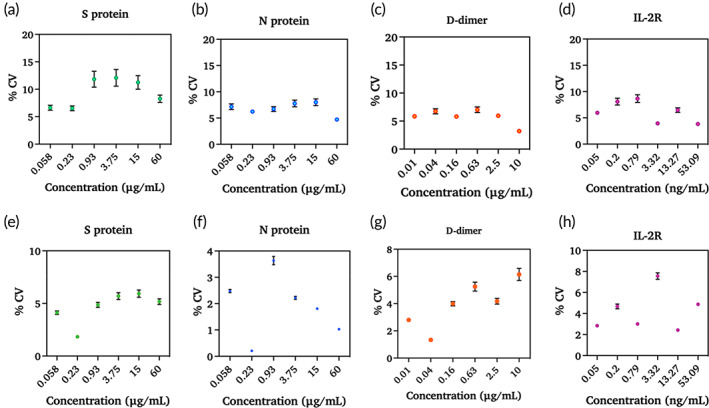
Inter and Intra assay variability tested for spike protein (a), (e), nucleocapsid protein (b), (f), D‐dimer (c), (g), and IL‐2R (d), (h).

The recovery rate and accuracy of the sensor for each biomarker were calculated and presented in Figure 4. Figure 4a–d shows the percent recovery for each biomarker at each concentration. Figure 4e–h shows the percent accuracy for each concentration measured. Both the accuracy and recovery range from 80% to 120%, that is, ± 20%. According to the Clinical Laboratory Standards Institute (CLSI), the sensor recovery should be within 20% of the actual concentrations. Inter‐ and intra‐assay reproducibility was estimated by calculating the percentage of covariance. Inter‐assay was performed between sensors, while intra‐assay refers to the repeated measurements within a sensor. The percentage of covariance was calculated as <15% for inter‐assay and < 10% for intra‐assay. This establishes that the results were consistent when using a single electrode for each concentration and the variations from the electrode to electrode are negligible. Therefore, by confirming the efficiency of the designed biosensor, it can be extensively applied to clinical utilization using a large number of samples. The results of inter‐assay (a)–(d) and intra‐assay (e)–(h) are shown in Figure 5 and Table S2. To the best of our knowledge, this is the first time that the reliable and simultaneous detection of spike protein, nucleocapsid protein, D‐dimer, and IL‐2R has been reported using the label‐free electrochemical impedance method.

### 3.4 Clinical validation of CoST sensor using clinical samples to distinguish healthy, mild, moderate, and severely affected patients

Validation studies provide information on the accuracy and reliability of the developed CoST sensor. Luminex was selected as a reference method to detect all the biomarkers used in the study. CoST sensor needs to exhibit a strong agreement with the reference method to implement this at a clinical site. Thus, the performance of the CoST sensor was validated with Luminex as a reference method to evaluate the quantification of biomarkers in patient plasma samples. A total of 40 patients (including healthy controls) were tested using both methods. Concentrations measured by the developed CoST sensor platform were well distributed over a wide dynamic range to distinguish healthy and COVID‐19 conditions. For testing with the CoST sensor, 100 μL of the sample was directly added to the CoST sensor without any further dilutions and sample preparations. Each of the CoST sensors was immobilized with antibodies for detecting all four biomarkers.

**FIGURE 6:**
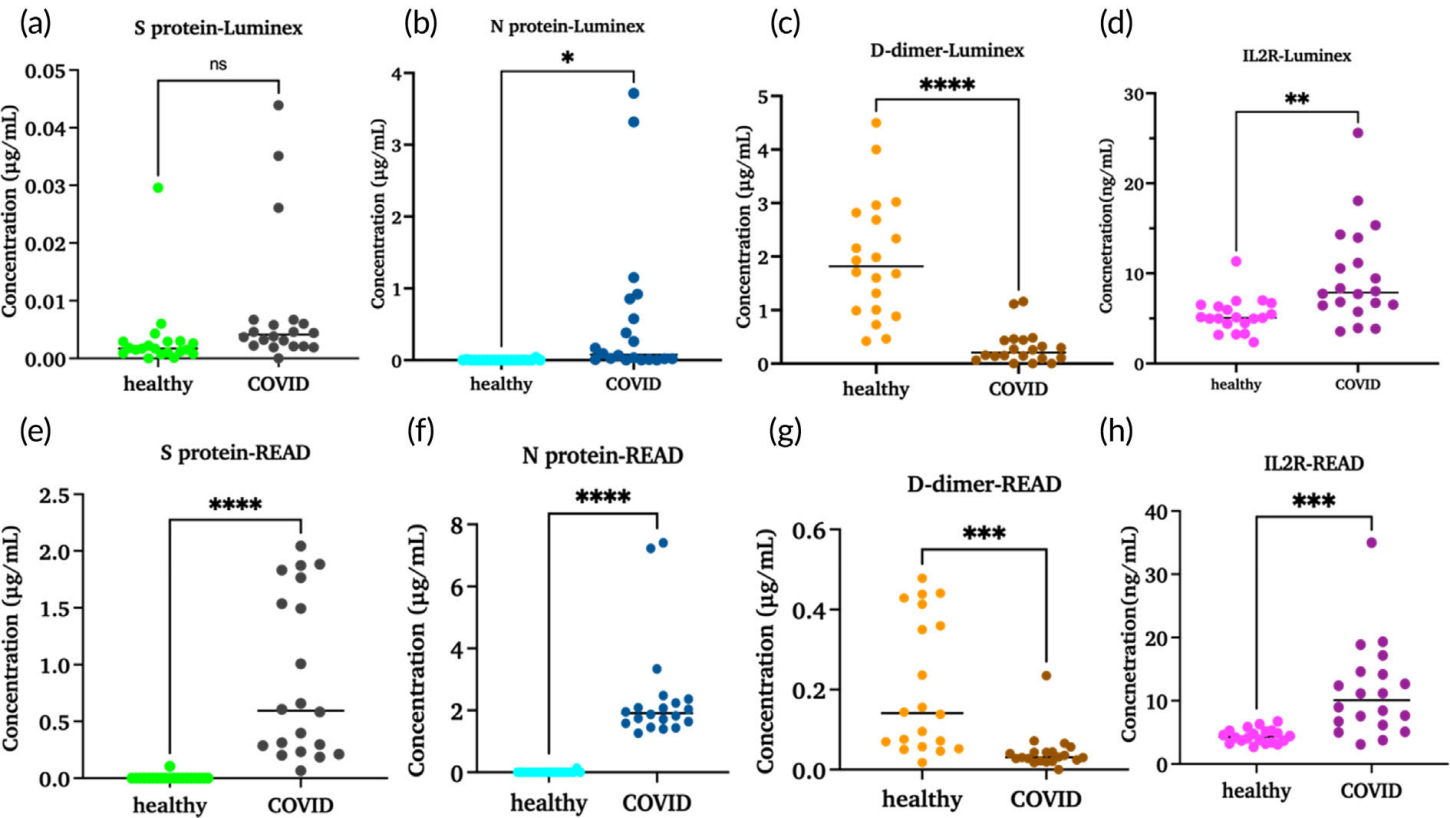
Healthy (*n* = 20) and COVID‐19 (*n* = 20) cohorts tested with Luminex (a)–(d) and CoST sensor (e)–(h).

**FIGURE 7:**
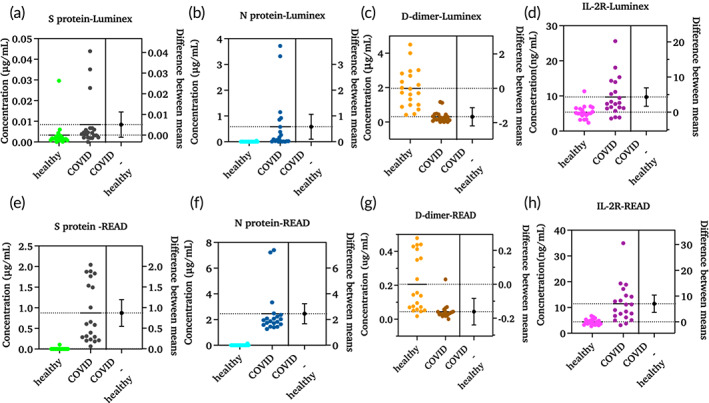
Estimation plots for defining mean difference between healthy and COVID‐19 cohorts using Luminex (a)–(d) and CoST sensor (e)–(h) (*n* = 20).

The CoST sensor was able to detect and measure the spike protein in all samples, unlike the Luminex method which was unable to measure it in one out of 40 clinical samples. The performance of the CoST sensor was better than Luminex in detecting spike protein in COVID‐19‐positive samples. Concentrations measured with Luminex were lower than concentrations measured with the CoST sensor in COVID‐19 cohorts. A significant difference with a *p*‐value of <0.0001 was observed with the CoST sensor, while Luminex showed a ns difference with a *p*‐value of 0.1 between the healthy and COVID‐19 cohorts for spike protein, as shown in Figure 6a, e. To measure the variation in spike protein concentration between healthy and COVID‐19 cohorts, an estimation plot was constructed. Using the CoST sensor, spike protein was measured in all COVID‐19 samples and showed a mean of 0.005 μg/mL for healthy samples and a mean of 0.87 μg/mL for COVID‐19 samples with a mean difference of 0.860.16 μg/mL between healthy and COVID‐19. Luminex data showed a mean value of 0.003 μg/mL for healthy samples and a mean value of 0.008 μg/mL for COVID‐19 samples with a mean difference of 0.005 ± 0.003 μg/mL between healthy and COVID‐19 (Figure 7a, e). This proves that the developed CoST sensor was able to quantify spike protein in all COVID‐19 samples, confirming its specificity for spike protein compared to Luminex. The mean value of spike protein concentration in the COVID‐19 cohorts is 170‐fold higher than the mean value of the healthy cohorts, indicating the characteristic importance of spike protein in COVID‐19 detection. The sensitivity of the CoST sensor was also found to be better than that of the standard reference method, based on the results in which Luminex showed a nonsignificant difference between healthy and COVID‐19 cohorts, whereas the CoST sensor showed a significant difference between healthy and COVID‐19 cohorts. Luminex showed higher spike protein levels than the CoST sensor in 19 of the 20 healthy cohorts. A significant difference with a *p*‐value of <0.0001 and a mean difference of 0.4337 ± 0.1055 μg/mL was observed between the CoST and Luminex methods in all 40 samples (Figure S3A, E).

Both healthy and COVID‐19 cohorts were analyzed for the presence of N protein using the developed CoST sensor as well as the Luminex reference method. Figure 6b shows the data obtained with the Luminex method and Figure 6f shows the data obtained with the CoST sensor. A significant difference was found between the healthy and COVID‐19 cohorts using both methods. The significant difference between healthy and COVID‐19 cohorts is high for the CoST sensor with a *p*‐value of <0.0001, while the Luminex method has a significant difference with a *p*‐value of 0.0198. We found a difference in quantification efficiency between the methods. The estimation plot data clearly shows the difference between the two methods for quantification of N protein with a mean difference of 0.9383 ± 0.3011 μg/mL (Figure S3F). The mean value of COVID‐19 cohorts is 0.5791 μg/mL with the Luminex method (Figure 7b) and 2.447 μg/mL with the CoST sensor (Figure 7f). This difference in mean value explains the efficiency of the CoST sensor in quantifying N protein in plasma samples. The mean value of nucleocapsid protein concentration in the COVID‐19 cohorts is 355‐fold higher than the mean value of the healthy cohorts, indicating the characteristic importance of nucleocapsid protein in COVID‐19 detection. A significant difference with a *p*‐value of 0.0026 was observed between the two methods for 40 samples (Figure S3B, F).

Literature has established that many COVID‐19 patients with a D‐dimer level >1.5 μg/mL showed an increased mortality rate compared to patients with a D‐dimer level <1.5 vg/mL. Therefore, 1.5 μg/mL is a cutoff value for predicting the mortality rate in COVID‐19 cohorts (51). All 40 samples, comprising both healthy and COVID‐19 cohorts, were tested using both the reference method and the developed CoST sensor. A significant difference was found between the healthy and COVID‐19 cohorts using both methods. CoST sensor showed a significant difference between healthy and COVID‐19 cohorts with a *p*‐value of 0.0002 whereas the reference method showed a *p*‐value of <0.0001 (Figure 6c, g). Surprisingly, the healthy cohorts had higher D‐dimer levels compared with the COVID‐19 cohorts when measured by both methods (Figure S3C, G). Luminex showed a mean value of 1.959 μg/mL for healthy cohorts, while the CoST sensor showed a mean value of 0.2059 μg/mL. Similarly, Luminex for COVID‐19 cohorts showed a mean of 0.3 μg/mL and the CoST sensor showed a mean of 0.04 μg/mL (Figure 7c, g). These remarkably low D‐dimer levels in COVID‐19‐19 cohorts compared with healthy individuals are due to several factors, as mentioned earlier. In the COVID‐19 cohorts, the medication history identified some information on age and medications that might have an impact on D‐dimer levels. Among 20 COVID‐19 cohorts, eight were taking medications that suppress blood clotting and control fibrinolysis, such as blood thinners and anticoagulants. These drugs are atorvastatin (five patients) and simvastatin (one patient), heparin (five patients), warfarin (one patient) and rivaroxaban (one patient), etc. Five of them also took a combination of two drugs. This could play a significant role in the fibrinolysis process in all cohorts who took these drugs. To accurately determine the effect of D‐dimers on COVID‐19 monitoring of their levels is required post‐infection. To date, there are very few electrochemical methods for monitoring D‐dimer levels, and these have not been explored in COVID‐19 infections. Another important factor affecting D‐dimer levels is the inflammatory status of each patient. Therefore, the effect of other inflammatory biomarkers in response to COVID‐19 infection is another influencing factor.

IL‐2R detection is important to measure T cell activation in infectious diseases. It is predicted to have clinical and prognostic significance on COVID‐19 disease progression. Both COVID‐19 and healthy cohorts were tested with Luminex and CoST sensors to quantify IL‐2R levels. COVID‐19 cohorts had very high IL‐2R levels compared with the healthy cohorts using both methods. A significant difference with a *p*‐value of 0.0019 was observed between healthy and COVID‐19 cohorts using the Luminex method, while the CoST sensor yielded a *p*‐value of <0.0001 (Figure 6d, h). A mean difference of 4.313 ± 1.296 ng/mL was observed between the healthy and COVID‐19 cohorts using the Luminex method, and the CoST sensor showed a higher mean difference of 7.303 ± 1.664 ng/mL. The median value measured using the CoST sensor for healthy cohorts was 4.413 ng/mL and for COVID‐19 cohorts are 11.44 ng/mL, showing the increase in IL‐2R levels in COVID‐19 (Figure 7d, h). A two‐tailed *t*‐test was performed to measure the significance between the two methods. A ns difference was found between Luminex and CoST sensors for quantifying IL‐2R in 40 samples. The mean concentration of IL‐2R with the Luminex was calculated to be 7.537 ng/mL and with the CoST sensor, it was 7.928 ng/mL in all 40 samples with a mean difference of 0.3912 ± 1.233 ng/mL between the methods (Figure S3D, H).

**FIGURE 8:**
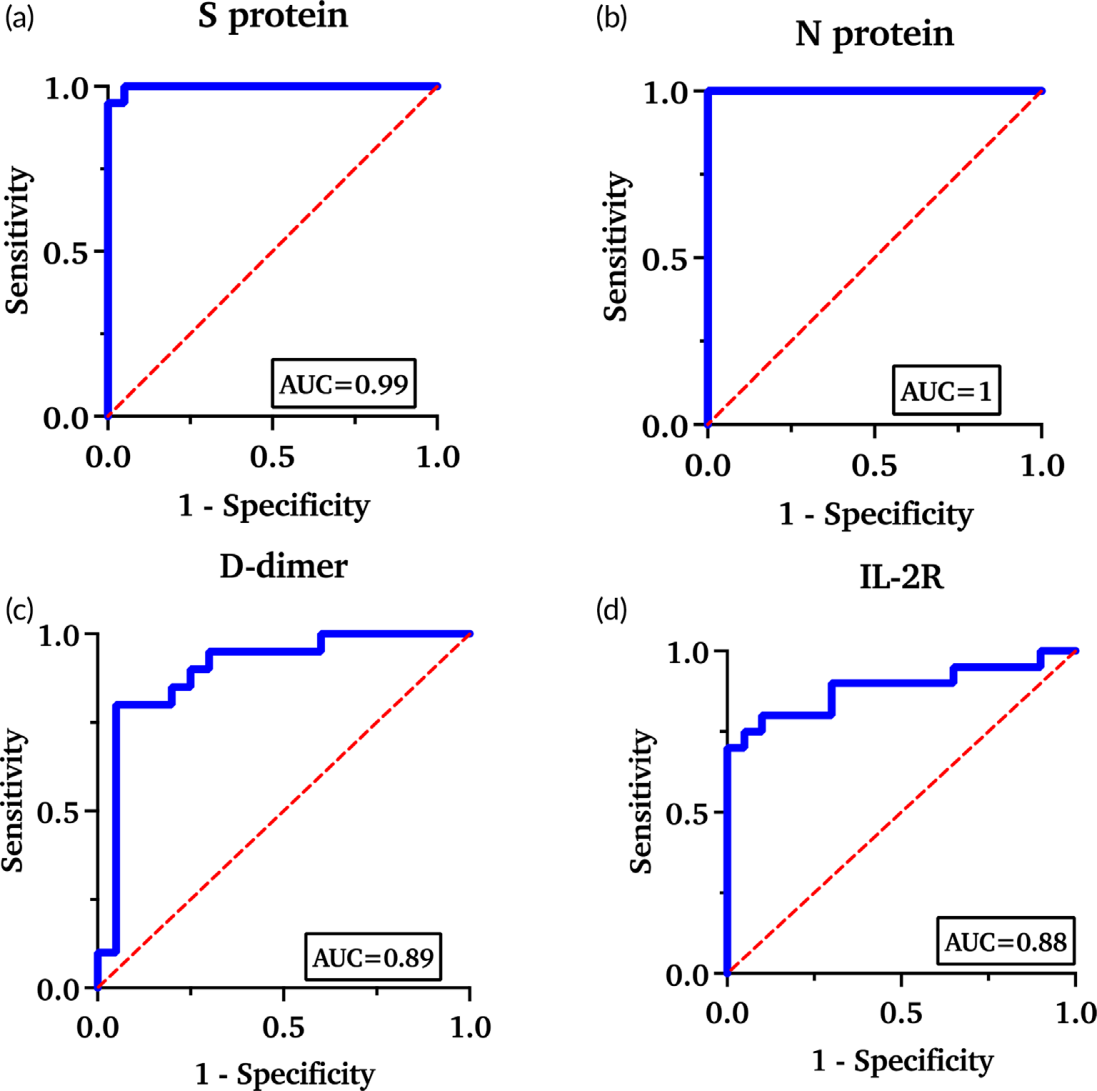
Receiver operating characteristic curve for all four biomarkers using CoST sensor (*n* = 20). Areas under the curve for the individual biomarker are labeled at the bottom right of each graph.

The difference between the two methods was evaluated using Bland–Altman analysis as shown in Figure S4A–D. The small mean deviation of 0.43, 0.93, 1, and 0.3 for S‐protein, N‐protein, D‐dimer, and IL‐2R, respectively, show the degree of proximity in measuring clinical samples by two methods. This proves the efficacy of the developed CoST sensor in clinical field trials. Further evaluation of the clinical utility of the CoST sensor was validated using a receiver operating characteristic curve. For any device, it is important to determine specificity and sensitivity to minimize false‐positive or false‐negative results. The diagnostic performance of the developed device can be determined by calculating the AUC. The specificity of the device is defined as the ability of the sensor to identify the target analyte, and its potential to discriminate non‐infectious samples. The ability of the device to detect the target analyte at very low concentrations defines its sensitivity. The AUCwas estimated using the ROC by performing the Wilson/Brown test at a 95% confidence level to evaluate the specificity and sensitivity of the developed CoST sensor for each of the target biomarkers. As shown in Figure 8a–d discriminative values were predicted based on the AUC value with a 95% confidence interval. N protein had the highest discriminatory value of 1, followed by S protein with an AUC of 0.99. D dimer and IL‐2R showed an AUC value of 0.89 and 0.88, respectively.

**FIGURE 9:**
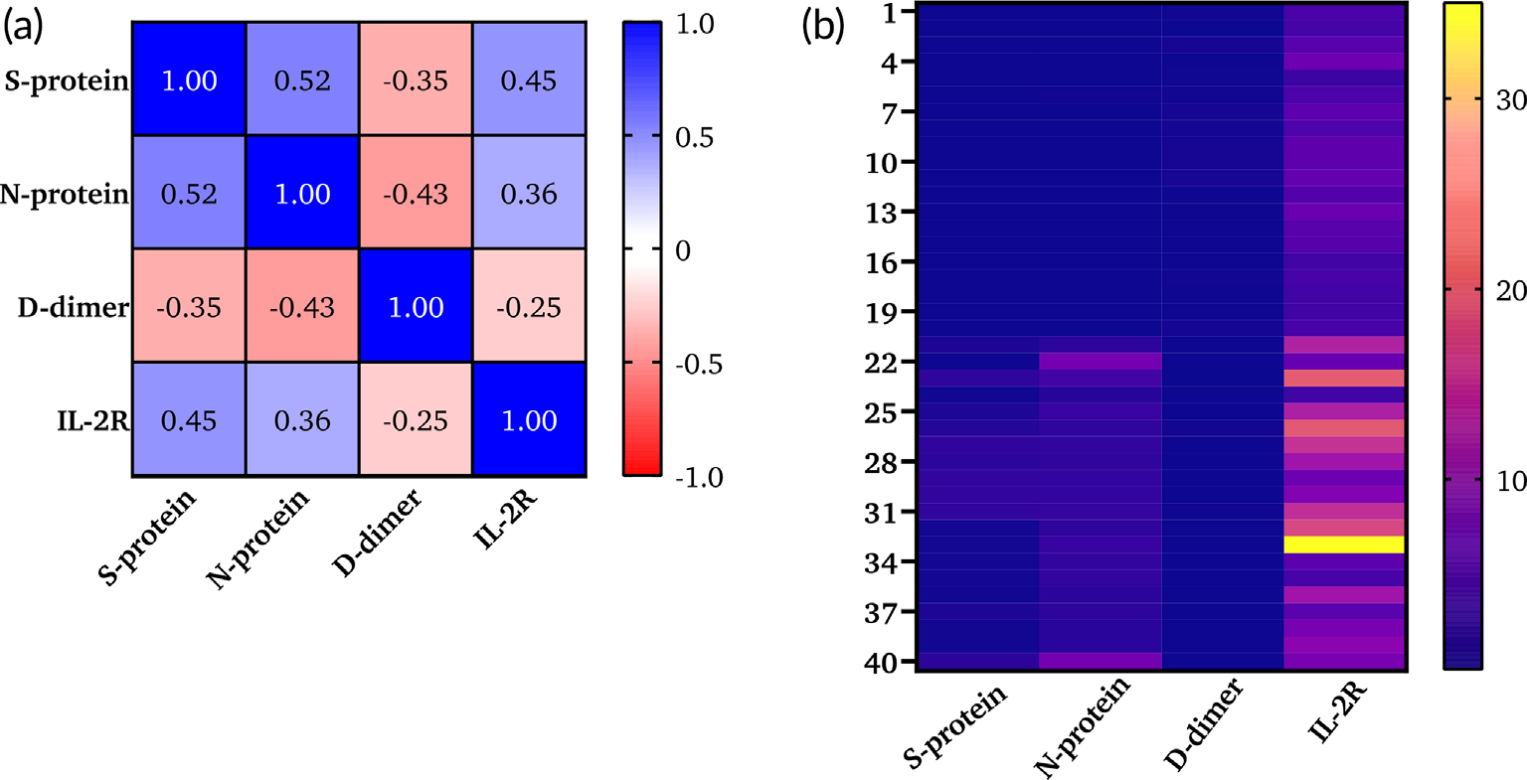
(a) Correlation analysis between all four biomarkers; (b) Heatmap plotted to evaluate the correlation between the biomarkers in all 40 samples (*n* = 40).

As shown in Figure 9, healthy samples had a very low IL‐2R, and COVID‐positive samples had a high IL‐2R, as indicated in the color bar in Figure 9b. D‐dimer showed no major differences between the healthy and COVID‐19 cohorts, as we described in the previous section. To assess the degree of correlation between biomarkers, a Pearson correlation matrix was constructed for all 40 samples (Figure 9a). N protein and S protein showed the highest correlation of 0.52, followed by S protein and IL‐2R with a correlation of 0.45. IL‐2R also showed a correlation of 0.36 with nucleocapsid protein. However, D‐dimer showed no positive correlation with all other three biomarkers. Descriptive statistical analysis was performed between healthy and COVID‐19 cohorts and presented in Table S3. The effect of other inflammatory markers on D‐dimer levels is also one of the factors besides the use of anticoagulant drugs to control the fibrinolysis process. The detection of biomarkers in plasma samples using the developed CoST sensor has several advantages compared to standard methods. We can extend the detection of these biomarkers to other biofluids. In this work, we have demonstrated a first‐of‐its‐kind host immune response monitoring point‐of‐care sensor that can detect all biomarkers simultaneously and quantify the levels of each biomarker within a few minutes with the equivalent of two drops of the body fluid. This study establishes that host immune response‐based stratification provides evidence‐based information that can be used to clinically manage the disease and hence arrest its progression. Through the CoST sensor we have found significant differences in the host immune response markers associated with disease severity namely IL‐2R, similarly, while D‐dimer is a useful marker to track disease severity progression, we have shown that the use of anti‐coagulants interferes with the expression profiles thus impacting its efficacy as a severity biomarker. The limited sample size limits the prediction of the risk rate depending on the biomarkers studied in this work. In addition, the measurement of a single time point is not sufficient to track the infection rate. However, the high sensitivity and specificity with very good accuracy in predicting the unknown concentrations are very helpful for clinicians to target the pathophysiology and severity of the disease. Apart from these limitations, the CoST sensor has other advantages as it has a wide dynamic range to evaluate the biomarker levels with high accuracy and specificity. It requires a small sample volume of approximately 100 μL, which is equivalent to 2–3 drops of blood, and provides rapid results. To date, there is no point‐of‐care device that can simultaneously measure the combination of S‐protein, N‐protein, D‐dimer, and IL‐2R and provide rapid results. We believe that the translational research presented here will be useful to the clinical community in rapidly assessing the risk category of COVID‐19 cohorts. Our lab is extensively working on the development of electrochemical immunosensors for infectious disease tracking and monitoring applications. This study focused on the detection of COVID‐19 and predict its severity based on D‐dimer and IL‐2R levels. Though our lab has previously worked on inflammatory biomarker detection in the context of infectious diseases, this is the first work where we have demonstrated COVID‐19 infection detection and severity prediction based on D‐dimer and IL‐2R levels.

## 4 CONCLUSIONS AND FUTURE PERSPECTIVES

In summary, we have developed a simple, highly selective, rapid, multi‐biomarker detection platform with low detection limits and high sensitivity. The detection limits of the sensor are 0.049 μg/mL, 0.003 μg/mL, 0.006 μg/mL, and 0.242 ng/mL for S and N proteins, D‐dimer and IL‐2R, respectively. The currently developed method can assure to provide a rapid diagnosis within 1 min and severity index using 100 μL of blood matrices. The developed CoST sensor platform will usher the scientific community toward a new class of medical device technology development. This portable, cost‐effective point‐of‐care device will contribute to the successful management of the COVID‐19 pandemic. As the developed COST sensor can successfully identify and quantify biomarkers, this portable ready‐to‐use biosensing platform can be linked to a smartphone‐based app to monitor patient health remotely. A risk‐indicating alert system can be set based on D‐dimer and IL‐2R levels, which helps the patients to rush to the hospital to take immediate treatment. Finally, the developed biosensor system is a promising tool and may be a suitable candidate for screening and surveillance of SARS‐CoV‐2 infection and post‐infection tracking which could lead to a breakthrough in public health management.

## AUTHOR CONTRIBUTIONS

**Sasya Madhurantakam:** Conceptualization (lead); data curation (lead); formal analysis (lead); investigation (lead); methodology (lead); project administration (lead); software (lead); validation (lead); writing – original draft (lead); writing – review and editing (lead). **Jayanth Babu Karnam:** Formal analysis (equal); resources (supporting); validation (supporting); writing – review and editing (supporting). **Sriram Muthukumar:** Conceptualization (supporting); methodology (supporting); project administration (supporting); resources (supporting); supervision (equal); validation (supporting); visualization (supporting); writing – review and editing (supporting). **Shalini Prasad:** Conceptualization (equal); funding acquisition (lead); investigation (equal); methodology (equal); project administration (equal); resources (lead); supervision (lead); writing – review and editing (supporting).

## FUNDING INFORMATION

The authors acknowledge that they received no funding in support of this research.

## CONFLICT OF INTEREST STATEMENT

Dr. Shalini Prasad and Dr. Sriram Muthukumar have a significant interest in EnLiSense LLC, a company that may have a commercial interest in the results of this research and technology. The potential individual conflict of interest has been reviewed and managed by The University of Texas at Dallas and played no role in the study design; in the collection, analysis, and interpretation of data; in the writing of the report, or in the decision to submit the report for publication.

### PEER REVIEW

The peer review history for this article is available at https://www.webofscience.com/api/gateway/wos/peer-review/10.1002/btm2.10566.

## Supporting information


**Data S1:** Supporting Information.

## Data Availability

All data generated or analyzed during this study are included in this published article (and its supplementary information files).
