# Supplementary material for: COVID severity test (CoST sensor)—An electrochemical immunosensing approach to stratify disease severity
Source: Bioeng Transl Med. 2023 Jul 11;8(5):e10566. doi: 10.1002/btm2.10566 (PMC10486328; doi:10.1002/btm2.10566)
Supplement: Supplementary file 1 — Data S1: Supporting Information. [file BTM2-8-e10566-s001.docx]

**S. Fig1: Immunoassay development steps for each biomarker (created Bioreder.com)**


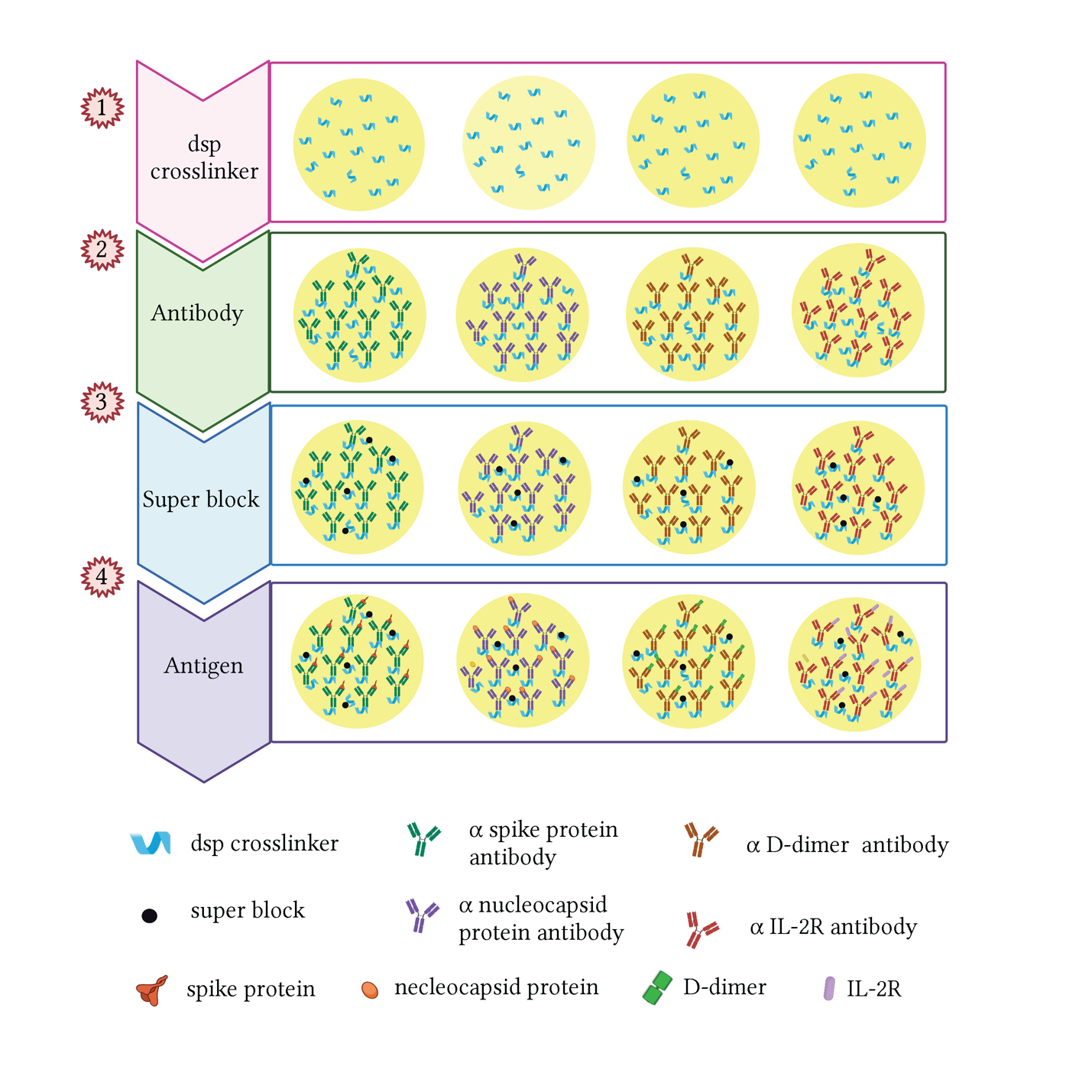


**S Fig2: FTIR spectrum of DSP and antibody functionalized surfaces (created Bioreder.com)**

**
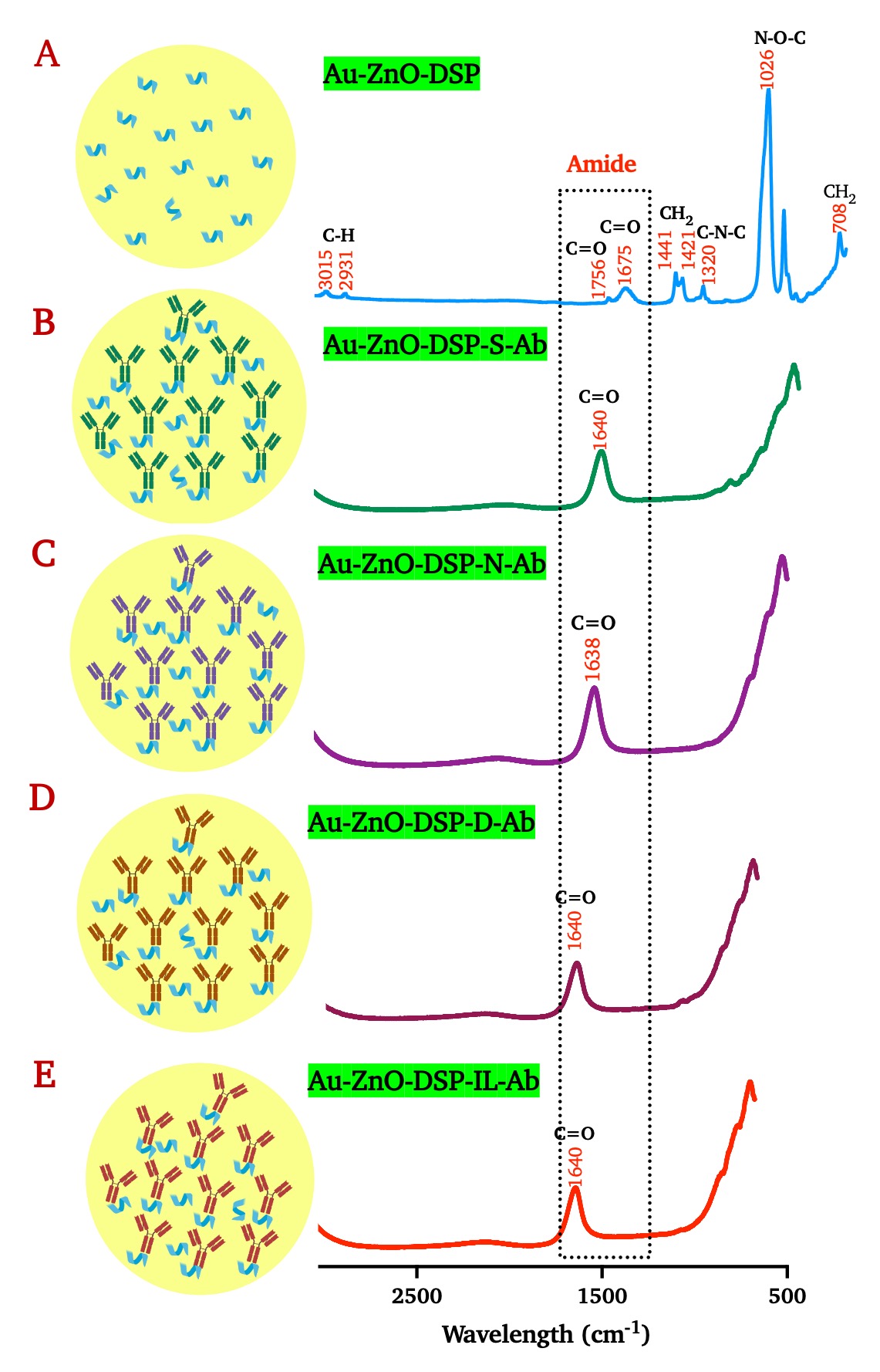
**

**S Fig 3: Comparing the data obtained from the developed CoST sensor and the reference Luminex method (A-D), estimation plot to measure the mean difference between CoST and Luminex methods (E-H).**


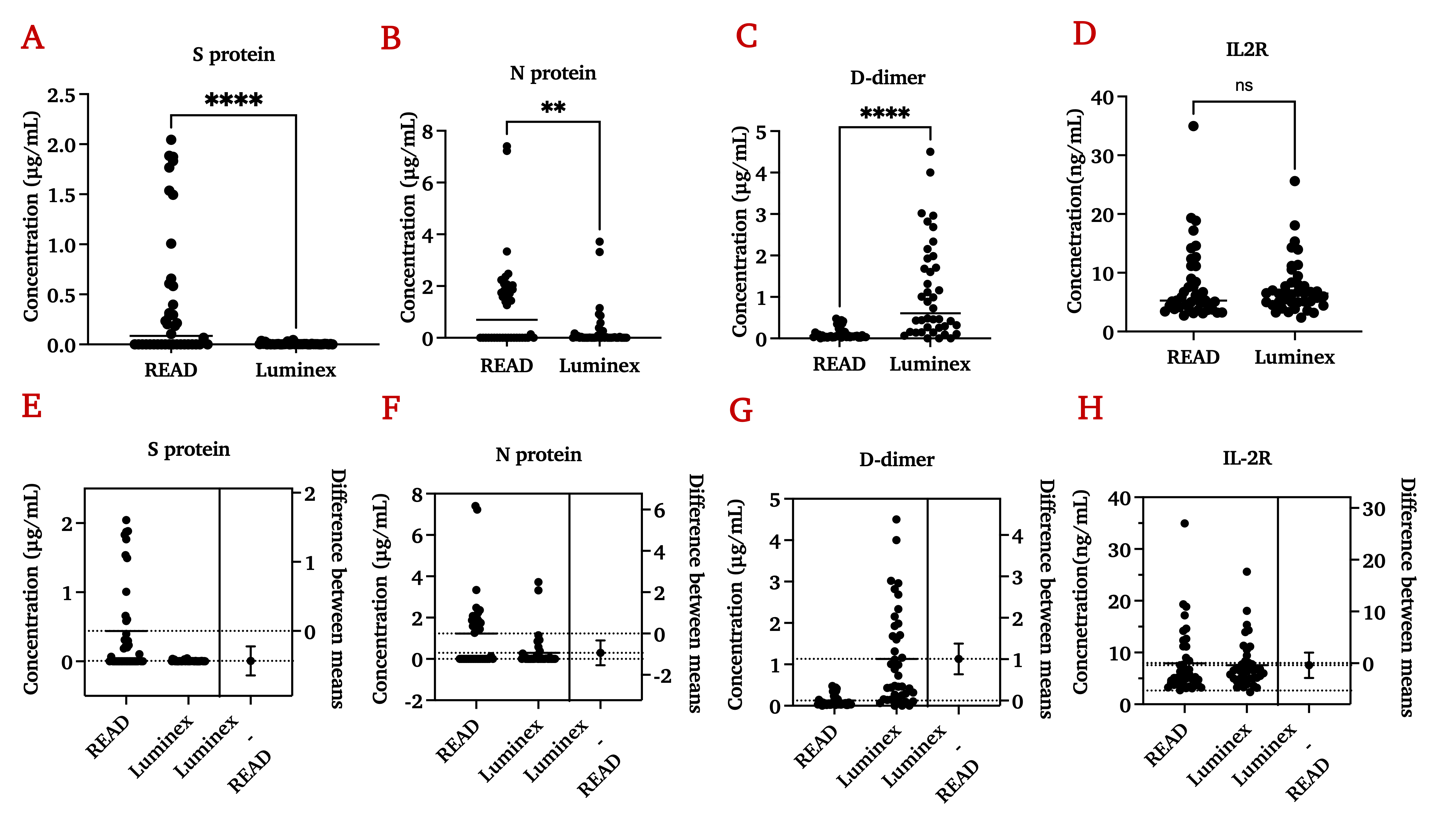


**S Fig 4: Bland-Altman analysis data for estimating the difference between the Luminex and CoST sensor using n=40 patient plasma samples (A-D)**

**S Table 1: Linear ranges along with LOD and LOQ and SST values for all the four biomarkers calculated using the developed CoST sensor.**

| **Biomarker** | **Linear range** | **LOD** | **LOQ** |
| --- | --- | --- | --- |
| S-protein | 0.058 – 60 μg/mL | 0.049 μg/mL | 0.065 μg/mL |
| N-protein | 0.058 – 60 μg/mL | 0.003 μg/mL | 0.021 μg/mL |
| D-dimer | 0.0009–10 μg/mL | 0.006 μg/mL | 0.016 μg/mL |
| IL-2R | 0.047–53.09 ng/mL | 0.242 ng/mL | 0.264 ng/mL |

**S Table 2: Inter-assay (A,B,C,D) and intra-assay (E,F,G,H) variability of the CoST sensor for all the 4 biomarkers at different concentrations**

| Biomarker | Concentrations | %CV (inter) | %CV (intra) |
| --- | --- | --- | --- |
| S-protein | 0.058 μg/mL | 6.61 | 4.11 |
|  | 0.23 μg/mL | 6.51 | 1.83 |
|  | 0.93 μg/mL | 11.73 | 4.90 |
|  | 3.75 μg/mL | 11.96 | 5.73 |
|  | 15 μg/mL | 11.16 | 5.91 |
|  | 60 μg/mL | 8.24 | 5.16 |
| N-protein | 0.058 μg/mL | 7.13 | 2.48 |
|  | 0.23 μg/mL | 6.20 | 0.21 |
|  | 0.93 μg/mL | 6.69 | 3.57 |
|  | 3.75 μg/mL | 7.74 | 2.19 |
|  | 15 μg/mL | 7.97 | 1.81 |
|  | 60 μg/mL | 4.70 | 1.03 |
| D-dimer | 0.01 μg/mL | 5.82 | 2.80 |
|  | 0.04 μg/mL | 6.72 | 1.34 |
|  | 0.16 μg/mL | 5.80 | 3.98 |
|  | 0.63 μg/mL | 7.00 | 5.30 |
|  | 2.5 μg/mL | 5.95 | 4.22 |
|  | 10 μg/mL | 3.20 | 6.21 |
| IL-2R | 0.05 ng/mL | 5.95 | 2.84 |
|  | 0.2 ng/mL | 8.06 | 4.66 |
|  | 0.79 ng/mL | 8.61 | 3.03 |
|  | 3.32 ng/mL | 3.93 | 7.35 |
|  | 13.27 ng/mL | 6.46 | 2.42 |
|  | 53.09 ng/mL | 3.81 | 4.76 |

**S. Table 3: Descriptive statistics calculated for both healthy (20) and COVID-19 (20) cohorts. Range, 10% and 90% percentile, median, confidence levels along with mean, standard deviation and standard error of mean are presented in the table for all 4-biomarkers**

|  | **S-protein** | | **N-protein** | | **D-dimer** | | **IL-2R** | |
| --- | --- | --- | --- | --- | --- | --- | --- | --- |
|  | **Healthy** | **COVID-19** | **Healthy** | **COVID-19** | **Healthy** | **COVID-19** | **Healthy** | **COVID-19** |
| **Number of values** | 20 | 20 | 20 | 20 | 20 | 20 | 20 | 20 |
|  |  |  |  |  |  |  |  |  |
| **Minimum** | 0.000048 | 0.065 | 0.000037 | 1.262 | 0.017 | 0.000009 | 2.70 | 3.11 |
| **25% Percentile** | 0.000049 | 0.246 | 0.000038 | 1.597 | 0.06 | 0.022 | 3.47 | 6.28 |
| **Median** | 0.000050 | 0.595 | 0.000039 | 1.908 | 0.141 | 0.030 | 4.29 | 10.07 |
| **75% Percentile** | 0.000051 | 1.708 | 0.000040 | 2.329 | 0.399 | 0.043 | 5.22 | 14.50 |
| **Maximum** | 0.104400 | 2.044 | 0.137300 | 7.401 | 0.478 | 0.234 | 6.73 | 34.97 |
| **Range** | 0.104400 | 1.978 | 0.137200 | 6.139 | 0.460 | 0.234 | 4.03 | 31.85 |
|  |  |  |  |  |  |  |  |  |
| **10% Percentile** | 0.000048 | 0.186 | 0.000037 | 1.4 | 0.046 | 0.017 | 3.10 | 3.87 |
| **90% Percentile** | 0.000054 | 1.883 | 0.000043 | 6.837 | 0.440 | 0.070 | 6.25 | 19.28 |
|  |  |  |  |  |  |  |  |  |
| **95% CI of median** |  |  |  |  |  |  |  |  |
| **Actual confidence level** | 95.86% | 95.86% | 95.86% | 95.86% | 95.86% | 95.86% | 95.86% | 95.86% |
| **Lower confidence limit** | 0.00005 | 0.2861 | 0.00004 | 1.63500 | 0.069 | 0.024 | 3.66 | 6.71 |
| **Upper confidence limit** | 0.00005 | 1.536 | 0.00004 | 2.23500 | 0.359 | 0.043 | 5.12 | 14.18 |
|  |  |  |  |  |  |  |  |  |
| **Mean** | 0.00527 | 0.8738 | 0.0069 | 2.454 | 0.2059 | 0.04424 | 4.413 | 11.44 |
| **Std. Deviation** | 0.02334 | 0.718 | 0.03069 | 1.726 | 0.1668 | 0.04795 | 1.11 | 7.358 |
| **Std. Error of Mean** | 0.00522 | 0.1605 | 0.00686 | 0.386 | 0.03729 | 0.01072 | 0.2482 | 1.645 |
